# A Human-Curated Annotation of the *Candida albicans* Genome

**DOI:** 10.1371/journal.pgen.0010001

**Published:** 2005-06-17

**Authors:** Burkhard R Braun, Marco van het Hoog, Christophe d'Enfert, Mikhail Martchenko, Jan Dungan, Alan Kuo, Diane O Inglis, M. Andrew Uhl, Hervé Hogues, Matthew Berriman, Michael Lorenz, Anastasia Levitin, Ursula Oberholzer, Catherine Bachewich, Doreen Harcus, Anne Marcil, Daniel Dignard, Tatiana Iouk, Rosa Zito, Lionel Frangeul, Fredj Tekaia, Kim Rutherford, Edwin Wang, Carol A Munro, Steve Bates, Neil A Gow, Lois L Hoyer, Gerwald Köhler, Joachim Morschhäuser, George Newport, Sadri Znaidi, Martine Raymond, Bernard Turcotte, Gavin Sherlock, Maria Costanzo, Jan Ihmels, Judith Berman, Dominique Sanglard, Nina Agabian, Aaron P Mitchell, Alexander D Johnson, Malcolm Whiteway, André Nantel

**Affiliations:** 1 Department of Microbiology and Immunology, University of California, San Francisco, California, United States of America; 2 Biotechnology Research Institute, National Research Council Canada, Montreal, Quebec, Canada; 3 Unité Postulante Biologie et Pathogénicité Fongiques, INRA USC 2019, Institut Pasteur, Paris, France; 4 Department of Stomatology, University of California, San Francisco, California, United States of America; 5 The Sanger Centre, Cambridge, United Kingdom; 6 Department of Microbiology and Molecular Genetics, Utah-Houston Medical School, Houston, Texas, United States of America; 7 Plate-Forme Intégration et Analyse Génomique, Institut Pasteur, Paris, France; 8 Unité de Génétique Moléculaire des Levures, Institut Pasteur, Paris, France; 9 School of Medical Sciences, University of Aberdeen, Institute of Medical Sciences, Foresterhill, Aberdeen, United Kingdom; 10 Department of Veterinary Pathobiology, University of Illinois at Urbana-Champaign, Urbana, Illinois, United States of America; 11 Institut für Molekulare Infektionsbiologie, Universität Wurzburg, Wurzburg, Germany; 12 Institut de Recherches Cliniques de Montreal, Montreal, Quebec, Canada; 13 Department of Medicine, Royal Victoria Hospital, McGill University, Montreal, Quebec, Canada; 14 Department of Genetics, Stanford University School of Medicine, Palo Alto, California, United States of America; 15 Department of Molecular Genetics, Weizmann Institute of Science, Rehovot, Israel; 16 Department of Genetics, Cell Biology, and Development, University of Minnesota, Minneapolis, Minnesota, United States of America; 17 Institute of Microbiology, University Hospital Lausanne, Lausanne, Switzerland; 18 Department of Microbiology and Institute of Cancer Research, Columbia University, New York, New York, United States of America; Yale University, United States of America

## Abstract

Recent sequencing and assembly of the genome for the fungal pathogen *Candida albicans* used simple automated procedures for the identification of putative genes. We have reviewed the entire assembly, both by hand and with additional bioinformatic resources, to accurately map and describe 6,354 genes and to identify 246 genes whose original database entries contained sequencing errors (or possibly mutations) that affect their reading frame. Comparison with other fungal genomes permitted the identification of numerous fungus-specific genes that might be targeted for antifungal therapy. We also observed that, compared to other fungi, the protein-coding sequences in the *C. albicans* genome are especially rich in short sequence repeats. Finally, our improved annotation permitted a detailed analysis of several multigene families, and comparative genomic studies showed that *C. albicans* has a far greater catabolic range, encoding respiratory Complex 1, several novel oxidoreductases and ketone body degrading enzymes, malonyl-CoA and enoyl-CoA carriers, several novel amino acid degrading enzymes, a variety of secreted catabolic lipases and proteases, and numerous transporters to assimilate the resulting nutrients. The results of these efforts will ensure that the *Candida* research community has uniform and comprehensive genomic information for medical research as well as for future diagnostic and therapeutic applications.

## Synopsis

*Candida albicans* is a commonly encountered fungal pathogen usually responsible for superficial infections (thrush and vaginitis). However, an estimated 30% of severe fungal infections, most due to *Candida,* result in death. Those who are most at risk include individuals taking immune-suppressive drugs following organ transplantation, people with HIV infection, premature infants, and cancer patients undergoing chemotherapy. Current therapies for this pathogen are made more difficult by the significant secondary effects of anti-fungal drugs that target proteins that are also found in the human host.

Recent sequencing and assembly of the genome for the fungal pathogen *C. albicans* used simple automated procedures for the identification of putative genes. Here, we report a detailed annotation of the 6,354 genes that are present in the genome sequence of this organism, essentially writing the dictionary of the *C. albicans* genome.

Comparison with other fungal genomes permitted the identification of numerous fungus-specific genes that are absent from the human genome and whose products might be targeted for antifungal therapy. The results of these efforts will thus ensure that the *Candida* research community has uniform and comprehensive genomic information for medical research, for the development of functional genomic tools as well as for future diagnostic and therapeutic applications.

## Introduction

*Candida albicans* is a commonly encountered fungal pathogen responsible for infections generally classed as either superficial (thrush and vaginitis) or systemic (such as life-threatening blood-borne candidiasis) [1,2]. Its life cycle has fascinating aspects that have generated great excitement over the last decade, with an influx of workers and new molecular techniques brought to bear on long-standing problems [3]. Topics of particular interest are the organism's capacity to shift into several different phenotypic states, some with distinct roles in infection, and its recently discovered capacity to mate, providing at least part of a sexual cycle, although population genetic studies indicate that it is still largely a clonal diploid population. Other special adaptations for infection include a battery of externally displayed proteins and secreted digestive enzymes; complex interactions with the host immune system normally keep *C. albicans* at bay as a minor part of the mucosal flora [1,4,5].

Here, we report a detailed annotation of the genome sequence of this organism, bringing the previously available raw sequence to a new level of stability and usability. The genome of *C. albicans* has previously been shotgun sequenced to a level of 10.9-fold coverage [6]. However the assembly of this sequence faced special difficulties because the organism is diploid but with little or no gene exchange in the wild. Thus homologous chromosomes show substantial divergence, and many genes are present as two distinctive alleles. This required that the assembly process be aware of the diploid status and be prepared to segregate reads into two alleles for any section of the genome. At the same time, the genome is rich in recently diverged gene families that are easily confused with alleles. This task was further complicated by the absence of a complete physical map of the *C. albicans* genome. Nevertheless, this arduous assembly process resulted in a dataset (assembly 19, with 266 primary contigs over eight chromosomes) that has already yielded a number of significant advances including the production of DNA microarrays [7], libraries of systematic gene knockouts [8], large-scale transposon mutagenesis [9], and the ability of many individual researchers to identify novel genes using bioinformatic tools [10]. Unfortunately, due to the mostly computational methods used in its development, the current genome assembly still contains a significant number of predicted genes that are fragmented, overlapping, or otherwise erroneous. As a consequence, different groups have been using different methods for the identification and classification of *C. albicans* genes, which has hindered communication and complicated comparisons between large-scale datasets.

Following the publication of these early functional genomics studies, it was realized that the needs of the *C. albicans* research community would be better served by a unified gene nomenclature. The results of this community-based effort were initially based on the version 19 computational assembly and preliminary annotation produced independently by various research groups. We used visual inspection of 11,615 putative coding sequences and various bioinformatic tools to refine the quality and description of each open reading frame (ORF).

In all, we provide unique identifiers, coordinates, names, and descriptions for 6,354 genes. With the exception of certain large gene families, we have not annotated the portion of the assembly 19 DNA that was set aside as secondary alleles, instead concentrating on the primary sequence that forms one haploid genome equivalent. Investigation of the identity and relative divergence of all alleles will be an important further project for the *C. albicans* genome, as will finishing and linking the small number of gaps that remain in the primary sequence. In addition, we describe a variety of gene families and we discuss insights into virulence. Finally, we use comparative genomics to point out a variety of additional insights that are illuminated by the high-quality annotation provided here. This project serves as a model for community-based annotation that could be applied by other research communities that wish to improve on automated sequencing pipeline output that may be available for their organisms of interest.

## Results/Discussion

### The Annotation Process

#### Compilation of *Candida* annotation data.

As detailed in Materials and Methods, we used assembly version 19 of the *C. albicans* genome [6] to identify 11,615 putative ORFs. These included genes encoding proteins greater than 150 aa as well as genes encoding smaller proteins of 50–149 aa that have a coding function greater than 0.5 as determined with a GeneMark matrix [11]. These ORFs were then compared to the set of 7,680 *C. albicans* ORFs defined by the Stanford Genome Technology Center (SGTC), thus permitting their classification using the same systematic identifiers of the format orf19.*n* [6]. The 3,936 novel ORFs without an orf19.*n* counterpart were assigned a new reference number of the format orf19.*n.i* where orf19.*n* is the five-prime closest (contig-wise) ORF defined by the SGTC and *i* is an integer that varies between one and the number of novel ORFs found in the orf19.*n* to orf19.(*n* + 1) interval. To simplify correlation with previously published data that use the orf6.*n* or earlier nomenclatures, we have produced a Web-accessible translation tool (http://candida.bri.nrc.ca).

Positional information for each ORF was merged with data from a variety of different sources, including the SGTC (http://www-sequence.stanford.edu/group/candida/index.html), CandidaDB (http://genolist.pasteur.fr/CandidaDB; [12]), the Agabian laboratory (http://agabian.ucsf.edu/canoDB/anno.php), and the Johnson/Fink laboratories [13], whose annotation data had been updated with a Magpie annotation [7]. This large dataset was then reformatted into EMBL-style files, thus allowing for input in the Artemis annotation software [14]. Volunteer annotators accessed a custom-made database to reserve and download EMBL files containing sequence and annotation data for each of the 266 DNA sequence contigs. To help in validating various and sometimes conflicting sources of information, translated protein sequences from putative *C. albicans* ORFs were compared to putative protein sequences extracted from five fungal genomes—*Saccharomyces cerevisiae* [15,16], *Schizosaccharomyces pombe* [17], *Neurospora crassa* [18], *Aspergillus nidulans* (*Aspergillus nidulans* Database, http://www-genome.wi.mit.edu/annotation/fungi/aspergillus/), and *Magnaporthe grisea* (*Magnaporthe grisea* Database, http://www-genome.wi.mit.edu/annotation/fungi/magnaporthe/)—as well as to the genomes of five other eukaryotes—*Arabidopsis thaliana* [19], *Drosophila melanogaster* [20], *Caenorhadbitis elegans* [21], *Mus musculus* [22], and *Homo sapiens* [23]—and the GenBank non-redundant (NR) protein database. Comparisons against the translated *C. albicans* genome were also performed to help identify overlapping genes and putative gene families.

**Figure 1:**
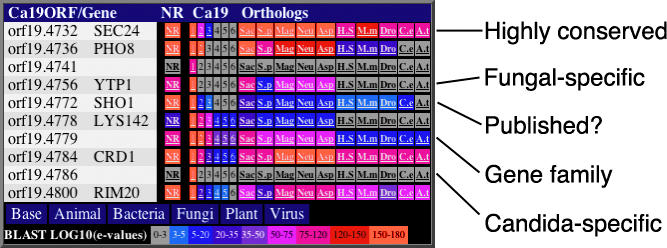
Visualization of Protein Sequence Similarities Sample from a Web page used by annotators of the *C. albicans* genome to visualize the significance of the best hit from whole-proteome BLASTP searches. Each putative ORF was compared to the NR database, the *Candida* ORF list itself (Ca19; showing results from the four top hits), and amino acid sequences from the proteomes of *S. cerevisiae* (Sac), *S. pombe* (S.p), *M. grisea* (Mag), *N. crassa* (Neu), *H. sapiens* (H.S), *M. musculus* (M.m), *D. melanogaster* (Dro), *C. elegans* (C.e), and *A. thaliana* (A.t). The BLASTP *e*-value from the top hit was converted to a color scale as indicated. Examples of *C. albicans* genes with interesting similarity patterns are indicated.

To help interpret such a large number of sequence comparisons, we organized sequence similarity data in a Web-accessible database using a novel visualization concept whereby we used a colorimetric display to indicate BLAST similarity, which was easy and rapid to scan visually (Figure 1). The annotators could thus rapidly determine which genes are potentially unique to *C. albicans* (e.g., orf19.4741 and orf19.4786), those that are members of gene families (e.g., orf19.4736 and orf19.4779), genes that only have homologs in fungal genomes (e.g., orf19.4756 and orf19.4778), or those with homologs in all eukaryotic genomes (e.g., orf19.4732 and orf19.4784). Finally, a strong hit against the complete NR database, but not in the other genomes (orf19.4772 and orf19.4800), allowed us to identify *C. albicans* genes that had already been described and submitted to the sequence databases prior to the publication of assembly orf19. Clicking on the relevant boxes opened an additional window containing the precompiled sequence alignments, thus permitting the validation of interesting observations. These visualization tools and the results of sequence comparisons are available at http://candida.bri.nrc.ca/candida/index.cfm?page=blast.

The coordinates and annotations for all 11,615 putative ORFs were thus verified, corrected, and (if necessary) rewritten by the annotators. We removed ORFs smaller than 300 bp with no significant sequence similarity to other genes, either within the *C. albicans* genome or in the sequence databases. In cases where two ORFs overlapped by more than 50%, the smallest gene was removed unless it showed even a slight sequence similarity to another gene in the sequence databases. In other cases, we encountered two, or more, contiguous ORFs that obviously were part of the same gene. These interruptions were usually due to unidentified introns or presumed sequencing errors. In these cases, we decided to merge the relevant gene fragments into a single entry. A total of 5,262 ORF entries were thus removed from the database, or merged with neighboring ORFs, leaving 6,354 confirmed genes. Sequence and/or annotation data can be obtained in Dataset S1 or at http://candida.bri.nrc.ca.

#### A nomenclature for *C. albicans* genes.

Following consultations with the *C. albicans* research community during the fifth and sixth American Society for Microbiology Conferences on *Candida* and Candidiasis, it was agreed that *C. albicans* gene names should follow the format established for *S. cerevisiae* [24]. Gene names consist of three letters (the gene symbol) followed by an integer (e.g., *ADE12*); the gene symbol should be an acronym for, or relate to, the gene function, gene product, or mutant phenotype. It is preferable that a given gene symbol have only one meaning, so that all genes using that symbol are related in some way, for instance, by sharing a function, participating in a shared pathway, or belonging to the same gene family. In addition, gene symbols that are used in *S. cerevisiae* gene names should retain the same meaning when used for *C. albicans* genes. The prefix ‘Ca' has sometimes been used on gene names to denote that a gene is derived from *C. albicans;* however, while the use of prefixes adds clarity to discussions of genes from different species that share a name (e.g., comparing Ca*URA3* to Sc*URA3*), the prefix is not considered part of the gene name proper. Finally, allele designations and deletion symbols should come after the gene name (*ICG1–8* and *icg1Δ* for example). For more details on genetic nomenclature, see the *Candida* Genome Database (CGD; [25]) Web page on this topic (http://www.candidagenome. org/Nomenclature.html).

Wherever possible, genes that are orthologous between *C. albicans* and *S. cerevisiae* should share the same name. We have provided 3,409 suggested names (in the SuggGene field of the EMBL files) for many *C. albicans* ORFs based on their orthology to *S. cerevisiae* genes; these are not yet considered the standard *C. albicans* gene names, but rather provide guidance for investigators wishing to name these genes. CGD assigns standard names to *C. albicans* genes for which there are published data (the PubGene field). The annotation contains 355 such entries. Generally, CGD considers the first published name in the correct format to be the standard name; common usage and uniqueness are also considered. All names that have been used for a gene are collected in CGD, regardless of their format, so that information from the literature can be traced to the correct gene. In the current annotation, additional published gene names have been placed in the Synonym field.

#### Public access to the data.

The complete annotation dataset, results of BLAST sequence similarity searches, and the identification of conserved protein domains can be obtained from our Web page (http://candida.bri.nrc.ca/). Furthermore, CGD (http://www.candidagenome.org), funded by the National Institute for Dental and Craniofacial Research of the National Institutes of Health, will curate the scientific literature and provide tools for accessing and analyzing the *C. albicans* genome sequence. In addition, CGD will act as a central repository for gene names and modifications, as approved by the *C. albicans* research community at the American Society for Microbiology *Candida* and Candidiasis meeting in Austin, Texas, in March 2004. CGD itself will not name *C. albicans* genes, but instead will act as a clearinghouse for the standard gene names and aliases, as the *Saccharomyces* Genome Database (SGD) does for the *S. cerevisiae* community. CGD hopes that researchers will follow CGD's gene nomenclature guidelines (see above) and keep CGD informed of any new gene names. Prior to publication, researchers may reserve a gene name, which will then become the standard name upon publication. Finally, the CandidaDB database (http://genolist.pasteur.fr/CandidaDB) [12], which has provided an annotation of the *C. albicans* genome sequence since January 2001, will be updated to take into account the complete annotation dataset and will continue to provide tools for accessing and analyzing the *C. albicans* genome sequence complementary to those available at the CGD and the Biotechnology Research Institute.

### Content and General Statistics

As detailed in Tables 1 and 2, we identified 6,354 genes in version 19 of the *C. albicans* genome assembly. This number is certain to change slightly with time as more data come to light. For instance, 80 of these genes are probably duplicates, having almost identical counterparts near the extremities of sequence contigs. Novel genes may also lie in unsequenced/unassembled gaps between the DNA sequence contigs. We identified 246 genes containing mutations or sequencing errors that result in a frameshift, or the insertion of a stop codon, that will have to be confirmed through resequencing. In the meantime, these elements have been joined as a single ORF entry and tagged with the entry “sequencing error?” inside their Note field. We have also identified 190 genes truncated at the ends of contigs, only 35 of which have an identical counterpart on a potentially overlapping contig. New information will be continuously integrated into the community data as it is submitted.

**Table 1:**
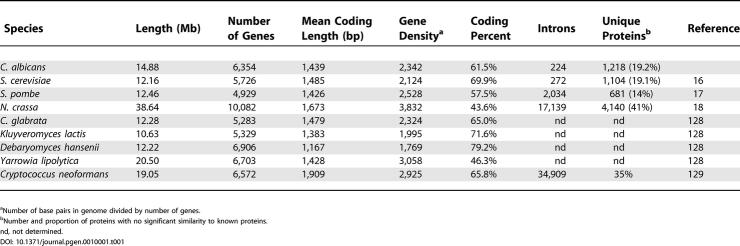
Features of Completed Fungal Genomes

^a^Number of base pairs in genome divided by number of genes.

^b^Number and proportion of proteins with no significant similarity to known proteins.

nd, not determined.

**Table 2:**
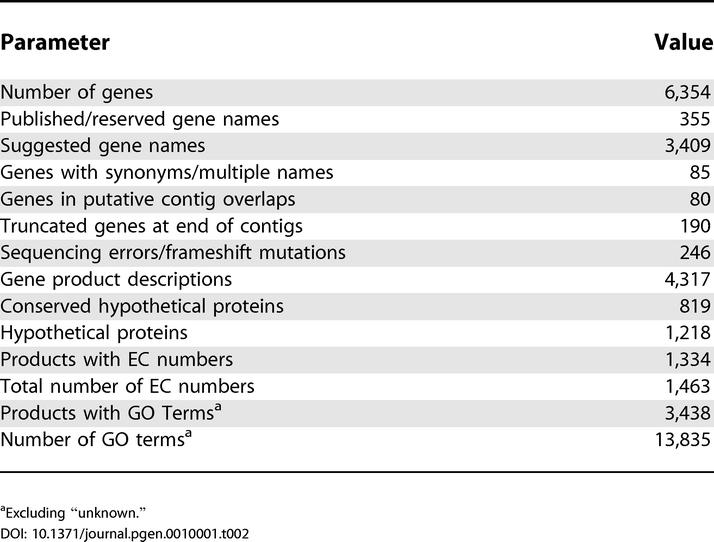
Statistics of the *C. albicans* Annotation

^a^Excluding “unknown.”

The mean protein coding length of 1,439 bp (480 aa) is almost identical to what has been observed in *S. cerevisiae* and *S. pombe,* while the gene density stands at one gene per 2,342 bp. Short descriptions for all gene products were provided by annotators, usually based on sequence similarity. A total of 1,218 (19.2%) genes encode unique proteins with no significant homologs in the sequence databases, a percentage almost identical to that observed in the current version of the *S. cerevisiae* annotation [16]. An additional 819 (12.9%) gene products exhibited significant similarities to other proteins of unknown function. Furthermore, we have provided Enzyme Commission (EC) numbers and Gene Ontology (GO) terms for 1,334 and 3,586 gene products, respectively.

#### Intron analysis.

There are 215 ORFs containing at least one intron, four of which have two introns, one gene (encoding the Hxt4p transporter) has three, and the *SIN3* gene has four. A total of 43 (20.2%) of these genes encode ribosomal proteins, 63 (29.6%) encode products with enzymatic activity, and 26 (12.2%) encode *trans*-membrane proteins involved in small molecule transport. We measured the relative position of introns in their host ORFs and observed that a significant proportion of them are located in the 5′ end of ORFs, with 32% of introns being located within the first 10% of the coding sequences. A survey of the distribution of introns in 18 eukaryotic genomes, including *S. cerevisiae* and *H. sapiens,* also indicated a similar bias in intron-poor genomes. It has been argued that this 5′ bias is an indication that introns are particularly difficult to remove by cDNA recombination, because of the high activity of these genes and paucity of full-length cDNA, and that this finding lends some support to the idea that introns are being lost more frequently than they are being gained in these lineages [26], although a more recent study of four fungal genomes suggests the presence of additional mechanisms [27].

We surveyed the intron phase distribution and found that *C. albicans* has 50.5%, 20.4%, and 29.1% of phase zero, one, and two introns, respectively. A similar result was observed in fungal, plant, and animal genomes [27,28], suggesting that a similar intron phase distribution may be present in ancient introns and that the intron loss has no preference selection on intron phases. Seventy out of 215 intron-containing ORFs have reciprocal best matches with *S. cerevisiae* genes that also contain introns. Among these 70 ORFs, 25 introns (35.7%) share the same position and the same phase. This suggests that these commonly positioned introns descended from a common ancestor, as suggested previously [29].

#### Analysis of protein domains.

Table 3 shows the most abundant protein domains that were identified in the *C. albicans* proteome. As a comparison, we also performed this analysis on the same ten eukaryotic proteomes that were used in the BLASTP sequence comparisons. Compared to the *S. pombe* and *S. cerevisiae* proteomes, the *C. albicans* proteome shows a slight increase in the abundance of leucine-rich repeats (IPR001611), some zinc finger transcription factors (IPR001138), esterases/lipases (IPR000379), and *trans*-membrane transporters for polyamines (IPR002293) and for amino acids (IPR004841). If the analysis is expanded to the other fungal proteomes, only the increased abundance in leucine-rich repeats appears to be unique to *C. albicans*.

**Table 3:**
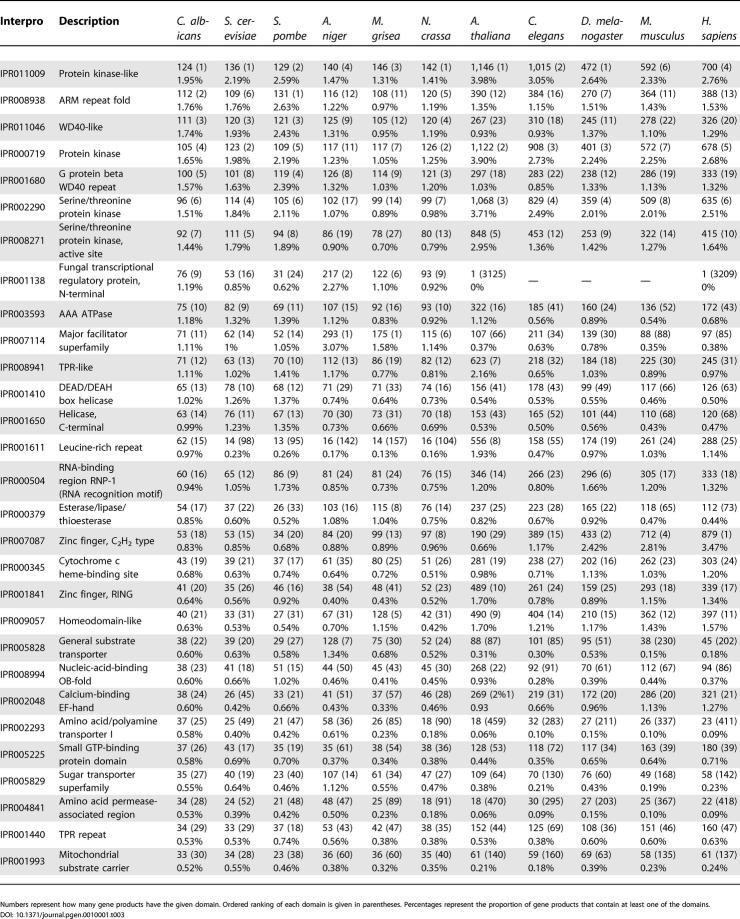
Number, Abundance Ranking, and Proportion of Gene Products Containing the Indicated Interpro Protein Domain in *C. albicans *and Other Eukaryotes

Numbers represent how many gene products have the given domain. Ordered ranking of each domain is given in parentheses. Percentages represent the proportion of gene products that contain at least one of the domains.

DOI: 10.1371/journal.pgen.0010001.t003

### Genome-Based Identification of Antifungal Targets

One of the main arguments supporting large-scale sequencing projects for fungal pathogens is the hope of finding novel antifungal targets, particularly those that are absent from the genome of their host. Table 4 shows a list of 228 *C. albicans* genes that have a very strong sequence homolog (based on a top hit BLASTP expect value (*e*-value) < 1e^−45^) in all five fungal genomes but no significant sequence similarity (best BLASTP *e*-value > 1e^−10^) to genes in the genomes of either humans or mice. For example, this list includes *FKS1,* which encodes a 1,3-beta-glucan synthase that is the target for the cell wall agents called echinocandins [30]. The list includes 46 gene products that are assumed to be located on the plasma membrane, 71 that are predicted to be involved in the transport of small molecules, and 21 that appear to be involved, directly or indirectly, with cell wall synthesis. Furthermore, 41 gene products have been associated with an EC number, indicating an enzymatic activity, with phospholipases being the most abundant. The roles and sites of action of these gene products suggest that they would be both accessible and theoretically amenable to inhibition by small molecules.

**Table 4:**
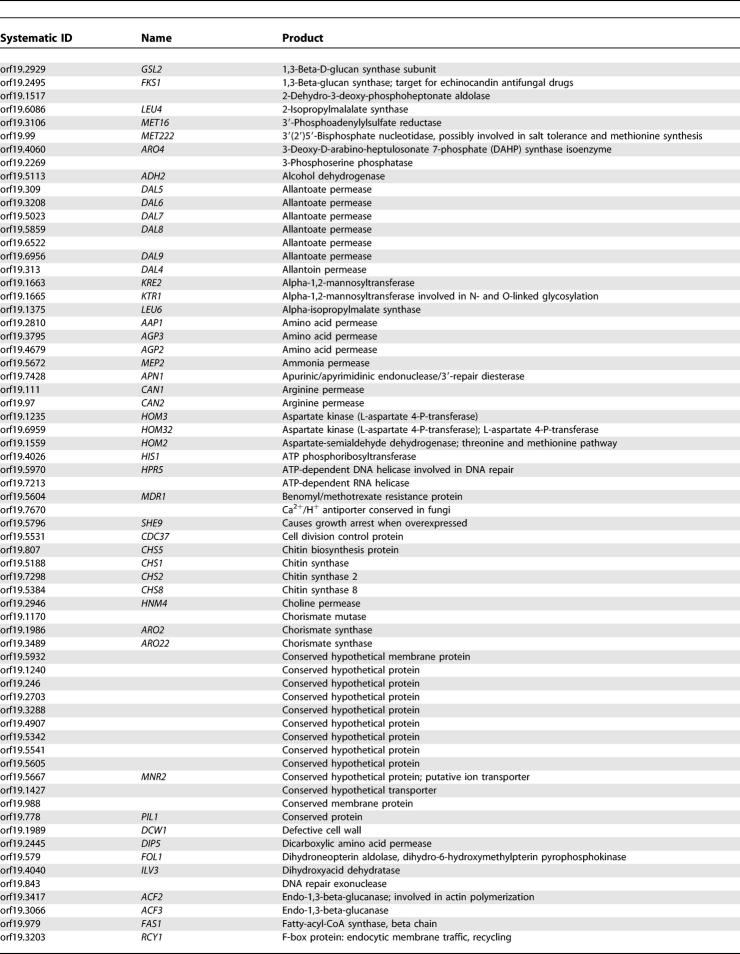
Genes from *C. albicans* with a Strong Homolog in the *S. cerevisiae*, *S. pombe, A. niger, M.* *gri*sea*,* and *N. crassa* genomes but Absent from the *H. sapiens* and *M. musculus* Genomes

**Table 4:**
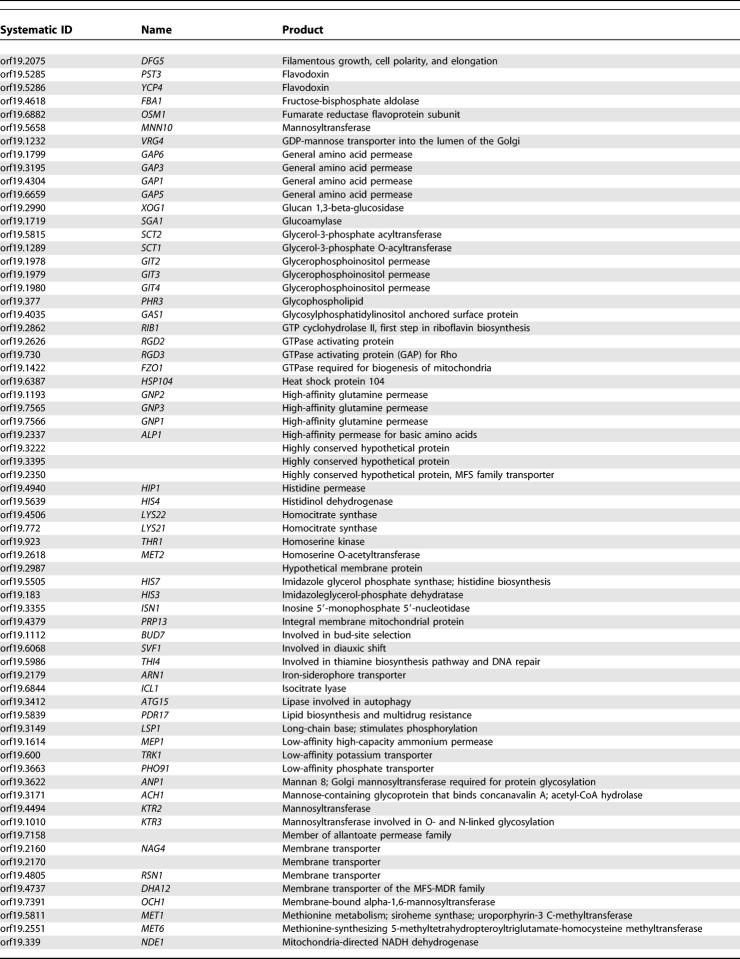
Continued

**Table 4:**
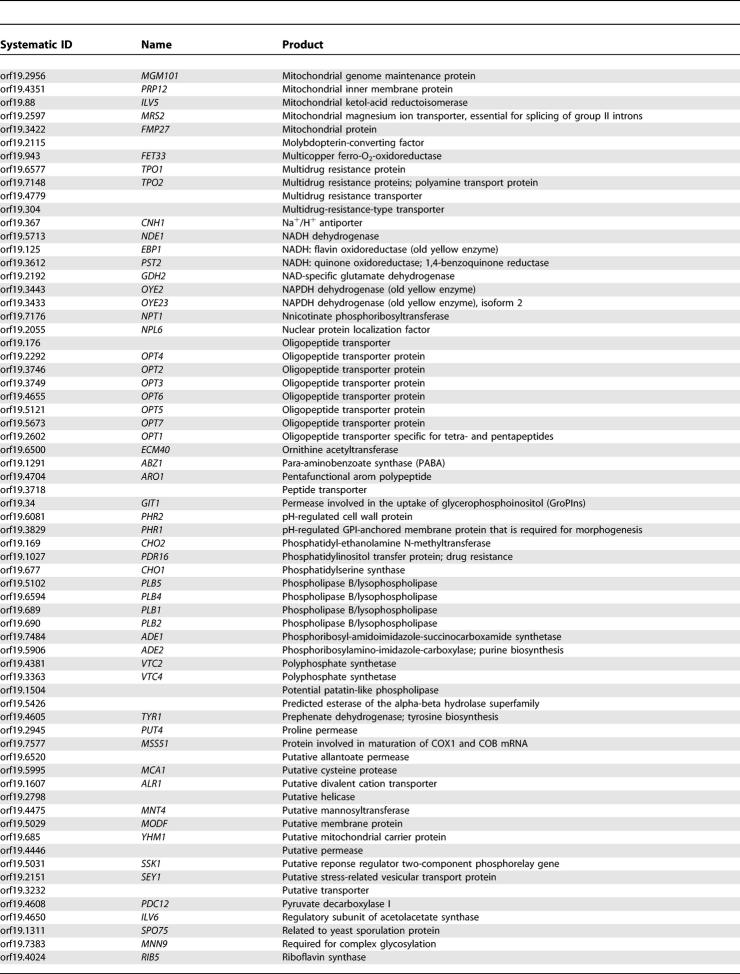
Continued

**Table 4:**
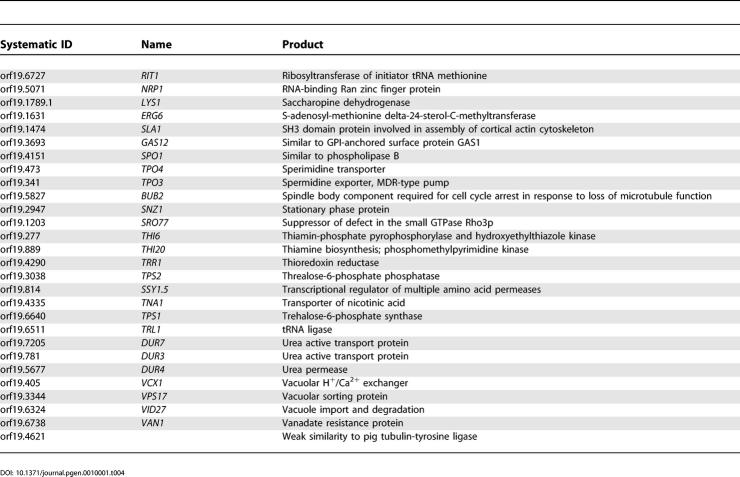
Continued

### Short Tandem Repeats

Short tandem repeats (STRs), also called short sequence repeats or microsatellite DNA, play an important role in evolution and have been used to characterize population variability. Although they can arise through DNA polymerase slippage and unequal recombination, whole-genome analysis has suggested that additional mechanisms for the control of STR production/correction remain to be identified [31–33]. Jones et al. [6] scanned the *C. albicans* genome for STRs of unit sizes between two and five and identified 1,940 trinucleotide repeats in their ORF sequences. To confirm that this high STR frequency is indeed a hallmark of the *C. albicans* genome, we used a statistical approach to measure repeat frequencies in four completed fungal genomes with an emphasis on STRs that affect protein sequences. We used randomized genome sequences to calculate the probability that each potential STR (including mutations that may arise following the amplification event) is nonrandom, and used only those with greater than 95% probability.

As can be seen in Datasets S2–S5 and Table 5, the STR frequencies in *C. albicans* and *N. crassa* are significantly greater than the frequencies observed in *S. cerevisiae* and *S. pombe*. Repeats that occur inside coding sequences are further characterized in Table 5. As would be expected, repeats with a modulo of three are more common in coding sequences, although we note that species with the greatest STR frequency have the smallest proportion of repeats that would break a reading frame. While coding sequence STRs in *C. albicans* and the other fungi most commonly encode for repeats of glutamine, asparagine, glutamic acid, and aspartic acid, we note that some of the repeats that are prevalent in *C. albicans* genes are distinct. Repeats of the ACT (threonine) and TCA (serine) codons are known to be especially rare in most taxa [31,33]. Correlating STR distribution with Gene Ontology annotations shows that a significant proportion of the *C. albicans* genes whose products are classified as DNA-binding proteins or cytoskeletal elements also contain STRs. Several gene products have been shown to play a role in the generation/correction of novel STRs in eukaryotes [34]. A comparison of the aa sequences of Rad51p, Rad52p, Mre1p, Hpr5p, and Pob3p from *C. albicans, S. cerevisiae, S. pombe,* and *N. crassa* did not reveal any significant correlation that could be associated with changes in the STR distribution. The high proportion of STRs in *C. albicans* genes argues that this organism would make a better model than *S. cerevisiae* for studying the creation and elongation of these elements that cause a variety of neuromuscular pathologies in humans. Our observations further indicate that future studies on STR frequency in eukaryotic genomes should include a broader spectrum of fungal genomes. The *S. cerevisiae* genome has been used as the fungal representative in comparative studies published to date [31–33].

**Table 5:**
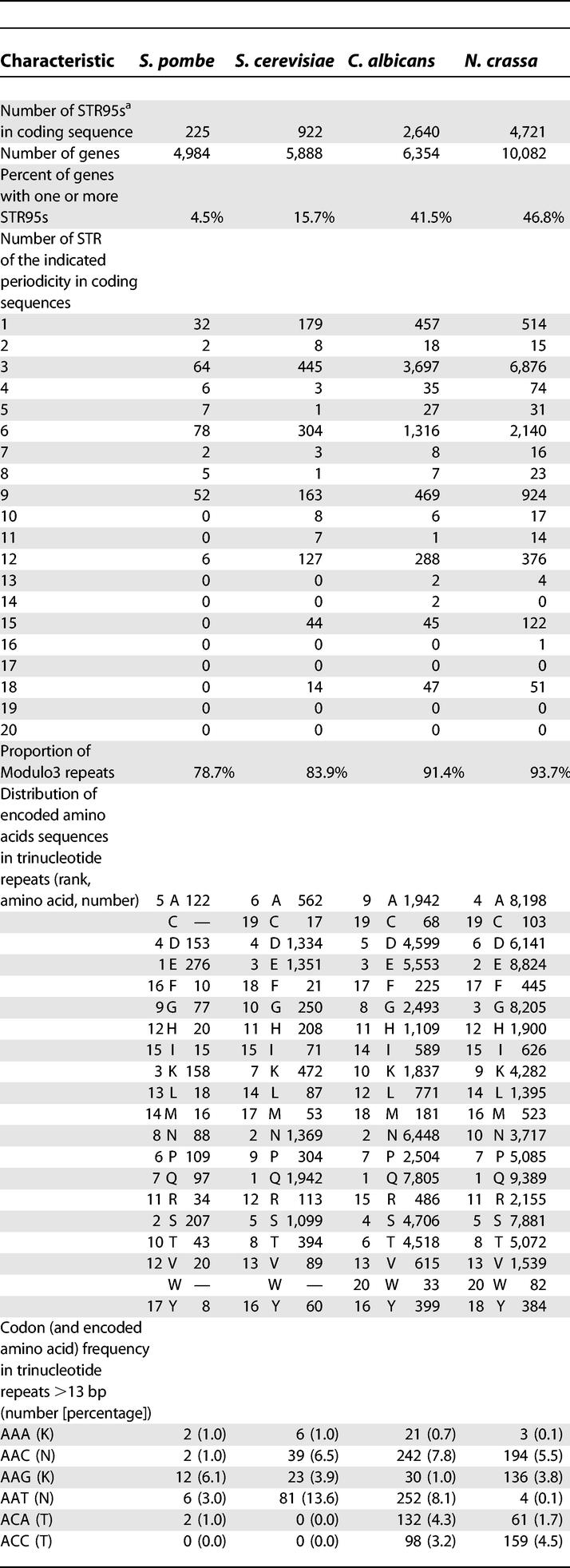
Frequency and Characteristics of Short Tandem Repeats in the Coding Sequences of Fungal Genomes

^a^STRs with a less than 5% chance of being random

**Table 5:**
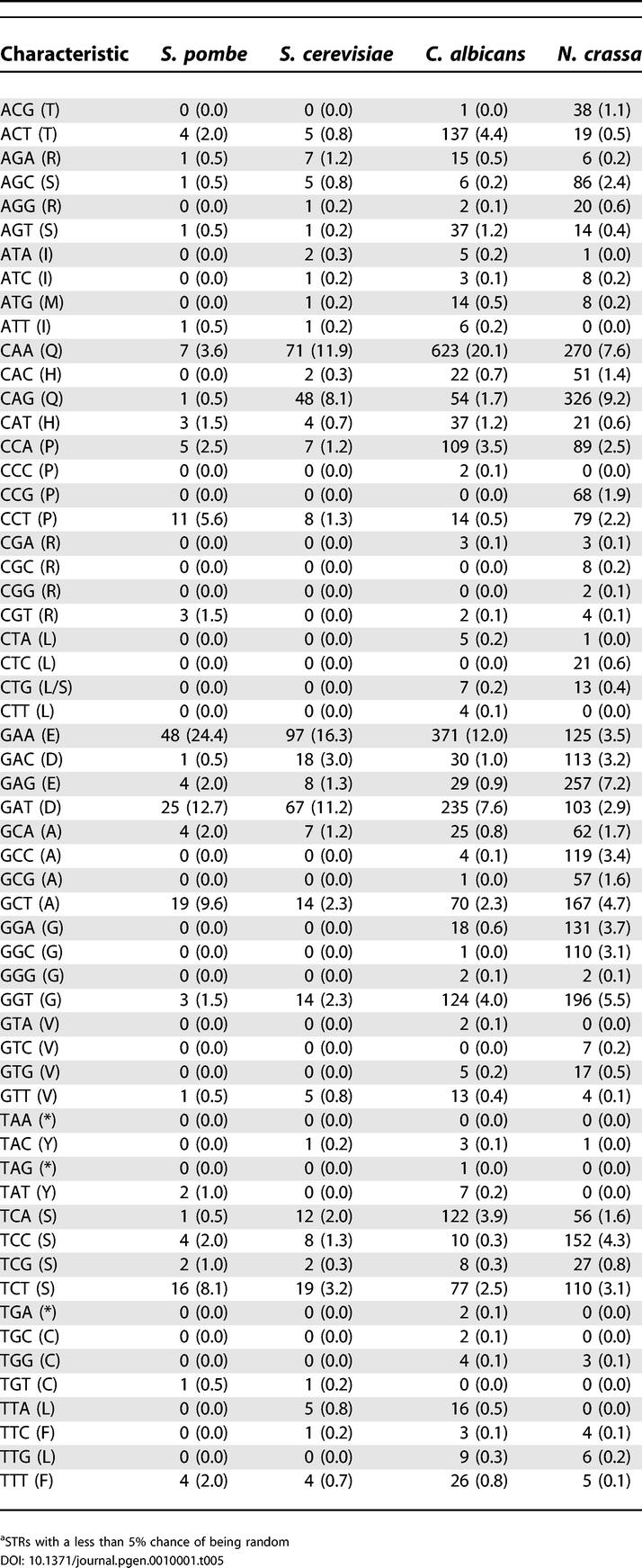
Continued

### Identification of Spurious Genes

**Figure 2:**
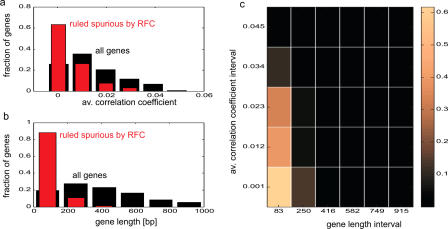
Identification of Spurious Genes Assessing criteria that identify candidate spurious genes in *S. cerevisiae,* using a reference set of known spurious genes [16]. (A) For every gene in *S. cerevisiae,* the average Pearson correlation coefficient with all other genes was calculated. Shown are histograms of the correlations associated with genes characterized as spurious in the reading frame conservation test ([16]; red) and all genes in the genome (black). (B) The distribution of gene lengths is shown for genes characterized as spurious (red) and for all genes of the genome (black). (C) Assessing the likelihood of being spurious as a function of gene length and correlation score. Shown is the proportion of spurious genes out of all genes whose length and correlation score fall into each of the intervals. The proportion is color-coded according to the color bar shown. *S. cerevisiae* genes with an ortholog in *C. albicans* were excluded from the analysis.

Some of the 6,354 predicted ORFs are likely to be spurious. We used data from *S. cerevisiae* to model an approach that combines gene length, gene homology, and gene expression data to search for spurious gene candidates. Theoretically, genes with no sequence similarity and with expression profiles that do not correlate with other known genes are much more likely to be spurious. In an earlier study, spurious genes in *S. cerevisiae* were identified by sequence comparison between four closely related yeast species [16]. Most did not have orthologs with other eukaryotes, were of short length, and had expression profiles that were not significantly correlated with those of other genes in the genome (Figure 2A and 2B). Combining both the criteria of sequence homology and expression correlation produced a list of *S. cerevisiae* candidate genes that was highly enriched for ORFs that were considered to be spurious based on the separate sequence comparison between the closely related species. We repeated this homology/expression/length analysis on genes of the *C. albicans* genome. *C. albicans* genes with an ortholog in other eukaryotes are assumed to be real and were excluded as candidates (510 of 513 *S. cerevisiae* genes ruled spurious by the reading frame conservation test [16] had no ortholog in *C. albicans*). In the above analysis, approximately 1,000 gene expression experiments were analyzed for *S. cerevisiae* [35], while approximately 200 currently available experiments were analyzed for *C. albicans* (see Materials and Methods). Table S1 includes a ranked list of the 349 *C. albicans* genes that are the most likely to be spurious.

### Multigene Families

Many putative and demonstrated virulence factors of *C. albicans* are members of large multigene families. Well-known examples of such families encode secreted aspartyl proteinases [36,37]), agglutinins [38], secreted lipases [39], high-affinity iron transporters [40], and ferric reductases [41]. Members of each of these families are differentially expressed as a function of the yeast–hyphae transition, phenotypic switching, or timing during experimental infection. Also, each of these families is large relative to the corresponding homolog or family of homologs in *S. cerevisiae,* leading to the concept that expansion of many *C. albicans* gene families may be an adaptation to a commensal lifestyle and may be, in part, responsible for *C. albicans*'s unusual ability to occupy a variety of host niches.

The sequencing of the genome provides an opportunity to survey the global occurrence and extent of multigene families as a first step in assessing their contribution to colonization and disease. We devised a purely computational method to define a comprehensive list of multigene families using NCBI-BLAST and custom Perl scripts. Each translated ORF in the annotated ORF set was compared to every other ORF in the set; if an ORF pair's BLAST alignment had an expectation value less than 1e^−30^ and a length greater than 60% of the length of the longer of the two ORFs, then the two ORFs were considered to be members of the same family. A transitive closure rule was applied to ensure that each ORF had membership in one and only one family. In all, 23% of the ORFs were members of families, a percentage comparable to that seen in other eukaryotes [18]. The approach yielded 451 families, with an average of 3.27 members each; 13 of the families have ten or more members, while the largest family has 39 members, consisting of proteins with possible leucine-rich repeat domains.

A striking difference between *C. albicans* and *S. cerevisiae* is the manner in which they acquire nutrients from the environment. In addition to the well-described secreted aspartyl proteinases, lipases, and high-affinity iron transporters, *C. albicans* possesses expanded families of acid sphingomyelinases (with four genes per haploid genome), phospholipases B (six genes), oligopeptide transporters (seven genes), and amino acid permeases (23–24 genes). Another striking difference is the emphasis by *C. albicans* on respiratory catabolism, as reflected in expanded families of peroxisomal enzymes. These include families of acyl-CoA oxidases (three genes), 3-ketoacyl-CoA thiolases (four genes), acyl-CoA thioesterases (three or four genes), fatty acid–CoA synthases (five genes), and glutathione peroxidases (four genes).

Additional families that may pertain to colonization or pathogenesis include those encoding the estrogen-binding protein OYE1 (seven genes), the fluconazole-resistance transporter FLU1 (13 genes), and the vacuolar protein PEP3/VPS16 (four genes), whose *Aspergillus* homolog is required for nuclear migration and polarized growth.

#### The ATP-binding cassette transporter superfamily.

The ATP-binding cassette (ABC) protein superfamily represents one of the largest protein families known to date among available genome sequences. These proteins share similar molecular architecture with the presence of at least one conserved ABC domain and the presence of membrane-spanning segments (transmembrane segments [TMSs]). The ABC domain typically contains Walker A and Walker B motifs and an ABC signature motif. The ABC domain and TMSs can be arranged in a duplicated forward (TMS_6_-ABC)_2_ or reverse (ABC-TMS_6_)_2_ topology, however “half size” ABC proteins also exist. As indicated in Table 6, the *C. albicans* genome contains at least 27 genes with ABC domains that include these topologies. These genes have been categorized, according to a classification established in *S. cerevisiae,* into six subfamilies (the MDR, PDR, MRP/CFTR, ALD, YEF3, and RLI subfamilies) [42]. The MDR, PDR, MRP/CFTR, and ALD subfamilies likely all encode transporter proteins, while the other subfamilies, YEF3 and RLI, generally lack TMSs and are considered as non-transporter ABC proteins. The *C. albicans* ABC proteins fall neatly into the categories developed for *S. cerevisiae,* and they are also present in approximately the same numbers (with the exception of the MRP/CFTR subfamily; see below). The predicted topology of each protein detailed in Table 6 is also largely comparable between the two yeast species. Among the 27 ABC proteins so far identified in *C. albicans,* the functions of only nine have been previously characterized. The largest group of known ABC transporters belongs to the *CDR* gene family, among which are *CDR1* and *CDR2,* two genes upregulated in azole-resistant clinical isolates that function in multidrug resistance [43–45]. *CDR3* and *CDR4* have been shown to function as phospholipid flippases and their expression is controlled by the white–opaque switching system [46,47]. Four MRP/CFTR-like transporters are present in *C. albicans,* and among them three show the NH_2_-terminal extension with additional transmembrane segments that is typical for many MRP-like transporters (see Table 6). For unknown reasons, homologs of additional members of this family, such as the *S. cerevisiae* genes *ScYBT1, ScNFT1,* and *ScVMR1,* are lacking in *C. albicans* [42,48]. Interestingly, the vacuolar MRP-like transporter encoded by *MLT1* has been implicated in virulence [49]. Since MRP/CFTR transporters are often involved in detoxification of heavy metals or xenobiotics, the presence or absence of discontinuous alleles of some ABC transporter genes (e.g., orf19.6383) may indicate strain differences in ABC transporter function and resulting susceptibility to environmental stresses. Most of the ABC transporter genes listed in Table 6 were given names through their closest homologs in *S. cerevisiae;* however, the functional assignments of these genes awaits further investigation.

**Table 6:**
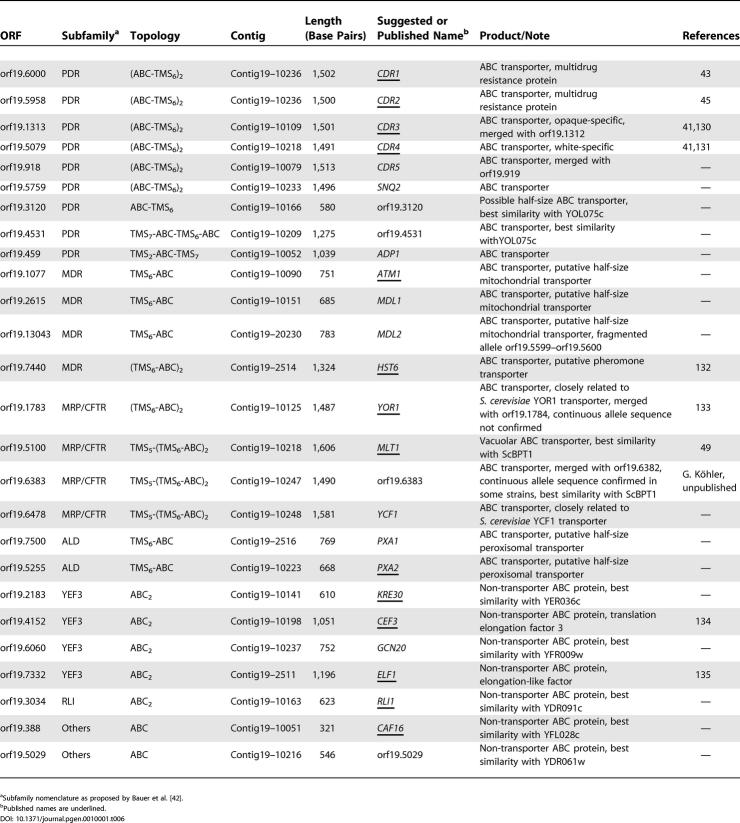
Genes Encoding Members of the ABC Transporter Family

^a^Subfamily nomenclature as proposed by Bauer et al. [42].

^b^Published names are underlined.

#### The ALS family.

The *ALS* genes encode large cell-surface glycoproteins that function in host–pathogen interactions [50,51]. The *ALS* genes are composed of three domains: a 5′ domain that is approximately 1,300 bp in length and relatively conserved in sequence across the family, a central domain composed entirely of tandemly repeated copies of a 108-bp sequence, and a 3′ domain of variable length and sequence that encodes a serine/threonine-rich portion of the protein [50]. Efforts to characterize the *ALS* genes started independently of the *C. albicans* genome project [38,52–54]) and were aided greatly by information that emerged as the genome sequencing effort progressed [55–57]. Table 7 lists the current ORFs that correspond to genes in the *ALS* family. The *ALS* family includes eight different genes [55], each with an extensive degree of allelic variability, sometimes within a given strain (Table 7) or across the wider population of *C. albicans* isolates [58–60]. Because of sequence assembly difficulties, mainly attributable to the length and repetitive nature of sequences within the *ALS* central domain, only three of *ALS* ORFs in this project are in agreement with *ALS* gene sequences derived independently of the genome project and reported in the literature (Table 7). The annotation effort described here did not edit the underlying assembly 19 sequence. However, gap sequencing that is presently being carried out and the production of a final genome assembly will correct these errors. Published *ALS* gene sequences can be found on the CGD Web site.

**Table 7:**
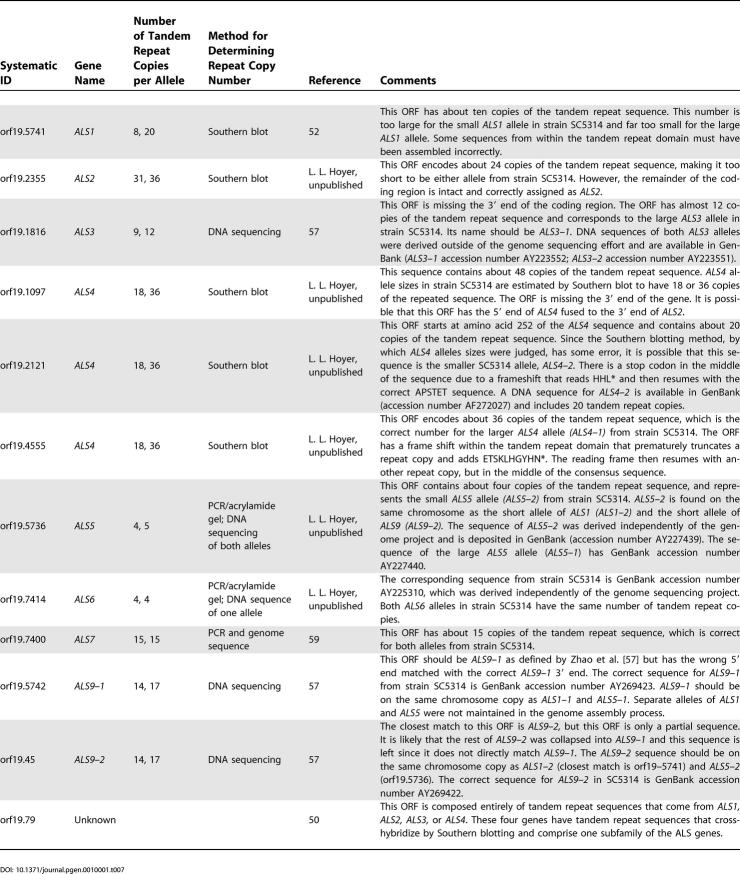
Assembly 19 ORFs That Correspond to ALS Genes

Assembly of the *C. albicans* genome sequence revealed the contiguous positions of *ALS5,* *ALS1,* and *ALS9* on Chromosome 6, which was verified by independent studies [57]. Additional testing revealed that, in SC5314, the large alleles of *ALS5,* *ALS1,* and *ALS9* occupy the same chromosome while the small alleles of each gene are found on the homologous chromosome [57]. But allelic variability and arrangement on homologous chromosomes will vary for each *C. albicans* strain. Allelic variation can be extreme for *ALS* genes, and is most commonly associated with the tandem repeat domain, although it is also present within other domains of the coding region [56,57,59]. Presenting the sequence of a single *ALS* allele, as done in these annotation data, loses the sense of allelic diversity that can have a significant effect on evaluation of ALS protein function. For example, testing the two *ALS3* alleles from strain SC5314 in a common adhesion assay format showed that the allele with more tandem repeat copies produced a protein with greater adhesive capability than the smaller allele [60]. Table 7 notes GenBank entries for *ALS* alleles from strain SC5314 that aid understanding of allelic diversity for the various *ALS* genes.

#### The *MEP* family.

Members of the *MEP* gene family encode ammonium permeases and, along with the *OPT* family described below, feature prominently in our list of fungal-specific genes. They thus represent potentially interesting targets for the development of antifungal drugs. Experimental evidence suggests that *MEP1* and *MEP2* encode the only specific ammonium permeases in *C. albicans*, since Δ*mep1* Δ*mep2* double mutants exhibited no detectable ammonium uptake and were unable to grow at ammonium concentrations below 5 mM [61], a phenotype that is similar to that of *S. cerevisiae* mutants deleted for all three ammonium permeases [62,63]. The third *C. albicans* gene, represented by orf19.4446, encodes a protein with much lower similarity to the other ammonium permeases of *C. albicans* and *S. cerevisiae* (approximately 44% to all proteins) but might encode an ammonium permease that is not expressed under the growth conditions used in these assays.

In addition to its role in ammonium transport, Mep2p also controls nitrogen-starvation-induced filamentous growth of *C. albicans*. Mutants in which only the *MEP2* gene was deleted grew as well as the wild-type strain at low ammonium concentrations but failed to filament under these conditions. This role of *MEP2* in filamentous growth of *C. albicans* at low ammonium concentrations is similar to the function of its counterpart *ScMEP2* in pseudohyphal growth of *S. cerevisiae* under limiting ammonium conditions [63]. However, in contrast to the latter, *MEP2* seems to have a much broader role in filamentous growth of *C. albicans* since Δ*mep2* mutants also had a filamentation defect when amino acids or urea instead of ammonium served as the limiting nitrogen source (J. Morschhäuser, personal communication).

#### The *OPT* family.

Oligopeptide transporters represent another group of fungal-specific surface proteins that transport peptides of four or five amino acids in length into the cell and together with the di- and tripetide transporters allow growth when peptides are the only available nitrogen source. This is presumably the position of *C. albicans* cells when they have invaded host tissues and are secreting their battery of peptidases and other catabolic enzymes. The founding member of the oligopeptide transporter gene family was *OPT1* from *C. albicans* [64]. Analysis of the *C. albicans* genome sequence as well as cloning of the corresponding genes demonstrated that *C. albicans* in fact possesses a large gene family encoding putative oligopeptide transporters. The *OPT* genes were annotated according to their decreasing similarity to *OPT1*. The *OPT2,* *OPT3,* and *OPT4* genes are highly similar to each other. The similarity of the remaining members of the family then drops considerably, but we have detected genes now named *OPT6,* *OPT7,* and *OPT8*. Deletion of the *OPT1* alleles in the *C. albicans* wild-type strain SC5314 resulted in increased resistance of the mutants to a toxic tetrapeptide, providing experimental evidence that Opt1p indeed functions as an oligopeptide transporter in *C. albicans* [65]. Preliminary observations indicate that at least the *OPT2* to *OPT5* genes also encode functional oligopeptide transporters (O. Reuß and J. Morschhäuser, unpublished data).

#### Zinc cluster transcription factors.

Proteins of the zinc finger superfamily represent one of the largest classes of DNA-binding proteins in eukaryotes. Several different classes of zinc finger domains exist that differ in the arrangement of their zinc-binding residues [66]. One of these domains, which appears to be restricted to fungi, consists of the Zn(II)_2_Cys_6_ binuclear cluster motif in which six cysteines coordinate two zinc atoms [67,68]. *S. cerevisiae* possesses 54 zinc cluster factors defined by the presence of the zinc cluster signature motif CX_2_CX_6_CX_5–16_CX_2_CX_6–8_C, which is generally located at the N-terminus of the protein. These proteins function as transcriptional regulators involved in various cellular processes including primary and secondary metabolism (e.g., Gal4p, Ppr1p, Hap1p, Cha4p, Leu3p, Lys14p, and Cat8p), pleiotropic drug resistance (e.g., Pdr1p, Pdr3p, and Yrr1p), and meiosis (Ume6p) [68,69]. Quite often, they bind as homo- or heterodimers to two CGG triplets organized as direct, indirect, or inverted repeats and separated by sequences of variable length [68,70]. A large proportion of these factors (50%) also contain a middle homology region (Fungal_*trans* in the Pfam Protein Families Database) located in the central portion of the protein that has been proposed to participate in DNA binding and to assist in DNA target discrimination [67].

Analysis of the *C. albicans* proteome using a combination of sequence analyses tools (SMART, Pfam, and PHI-BLAST) allowed us to identify 77 binuclear cluster proteins. These factors are characterized by the presence of the zinc cluster signature motif CX_2_CX_6_CX_5–24_CX_2_CX_6–9_C generally located at the N-terminus of the protein (72 out of 77) and with a spacing between cysteines 3–4 and 5–6 slightly different from the *S. cerevisiae* motif. As observed in *S. cerevisiae,* a large proportion of the *C. albicans* factors also contain a middle homology region (29 out of 77). To our knowledge, only six of the *C. albicans* zinc cluster genes have been characterized in detail, including *SUC1,* involved in sucrose utilization [71], *FCR1,* implicated in pleiotropic drug resistance [72], *CWT1,* required for cell wall integrity [73], and *CZF1,* *FGR17,* and *FGR27,* involved in filamentous growth [9,74]. The functions of many uncharacterized *C. albicans* zinc cluster factors (approximately 20%) can be inferred from the fact that they display high levels of sequence similarity (top BLASTP *e-*value ≤ 1e^−20^) with the products of *S. cerevisiae* genes with a known function. In the case of *GAL4,* however, the *C. albicans* homologous ORF identified (orf19.5338) encodes a significantly smaller protein (261 aa) than *S. cerevisiae* Gal4p (881 aa), lacking the C-terminal two-thirds of the protein that contains one of two transcriptional activating domains, and must therefore have a somewhat different function. Approximately half of the *C. albicans* zinc cluster genes do not appear to have homologs in *S. cerevisiae* (using a BLAST cutoff of < 1e^−20^) and are therefore likely to participate in processes specific to *C. albicans*. Finally, it is noteworthy that many of the zinc cluster factors known to be involved in pleiotropic drug resistance in *S. cerevisiae,* such as Pdr1p, Pdr3p, Yrr1p, Yrm1p, Rds1p, and Rdr1p, do not appear to possess close structural homologs in *C. albicans*. Since pleiotropic drug resistance is frequently observed in *C. albicans,* it is likely that this organism possesses functional homologs of these genes or other novel processes that remain to be identified.

### Lipid and Amino Acid Metabolism

Some of the *C. albicans* ORFs that do not have clear homologs in *S. cerevisiae* but do have homologs in other fungi, bacteria, and/or vertebrates encode catabolic enzymes, oxidoreductases, and proteins involved in environmental sensing pathways. The list of genes that *C. albicans* does not share with *S. cerevisiae* is skewed towards enzymes involved in the catabolism of fatty acids and ketone bodies in the peroxisome. There are also numerous oxidoreductases, some of which may be involved in activating hydrophobic organic compounds as a prelude to their oxidative degradation. This metabolic arrangement may reflect, in part, the state of the common ancestor with *S. cerevisiae,* as also reflected in *Yarrowia lipolytica, C. antartica, C. rugosa, C. tropicalis, C. maltosa,* and *C. deformans,* which are model organisms in the study of lipases and alkane oxidation for industrial purposes. It is worth mentioning, however, that the genus *Candida* arose originally to identify fungi that were unclassifiable, asexual, and ascomycetous—properties that appear to correlate with parasitism and the presence of catabolic gene families, such as lipases and alkane-assimilating cytochrome P-450 enzymes. Beta-oxidation in fungi is predominantly peroxisomal, and the number of enzymes participating in the process is greater in *C. albicans* than in *S. cerevisiae*. *C. albicans* also encodes a related ethanolamine kinase (orf19.6912), a malonyl-CoA acyl carrier protein acyltransferase (MCT1), and an enoyl-CoA hydratase (orf19.6830) not found in *S. cerevisiae*. Further supplying substrates for oxidation are several enzymes encoded by *C. albicans* that participate in the degradation of asparagine (asparaginase; orf19.3791), cysteine (cysteine dioxygenase [CDG1] and cysteine sulfinate decarboxylase [orf19.5393]), valine (3-hydroxyisobutyrate dehydrogenase [orf19.5565]), and arginine (orf19.3498). Other catabolic enzymes come as a surprise in that they may relate to the scavenging of unsuspected carbon sources. *C. albicans* encodes three D-amino acid oxidases (IFG3, DAO1, and DAO2) whose substrates might be derived from bacterial cell walls, various oxidoreductases whose substrates are likely to be aromatic and aliphatic compounds not used by the host, a pathway consistent with omega oxidation of fatty acids (which would convert alkanes into alpha-omega diols, fatty acids, and dicarboxylic acids), and a benzene desulfurase (orf19.3901).

Acetyl-CoA generated in the peroxisome is transferred to the mitochondrion, where the most notable difference from *S. cerevisiae* is the presence of a respiratory Complex I, which can now largely be reconstructed based on sequence similarity to components found in other organisms. The importance of Complex I in the biology of *C. albicans* is inferred from the observation that deletion of one of its subunits results in a defect in filamentation [75] and the observation that subunit 49 is essential for vegetative growth [8]. An additional difference is the presence of two alternative oxidases that may be involved in protection against oxidative stress [76]. Thus, it is not yet clear whether the omnivorous catabolic capacity of *C. albicans* reflects its heritage and role as a fungal saprophyte aiding organic decomposition, or whether these capacities have been elaborated and tuned in response to the specific problem of consuming mammalian host cells.

#### Phospholipases.

Depending on the site of attack, phospholipases are classified as phospholipase A, B, C, or D. Phospholipase A enzymes hydrolyze the 1-acyl ester (PLA_1_) or the 2-acyl ester (PLA_2_) of phospholipids. In fungi, phospholipase B enzymes hydrolyze both acyl groups and often also have lysophospholipase activity, removing the remaining acyl moiety on lysophospholipids [77]. Phospholipase C and phospholipase D enzymes are phosphodiesterases that cleave the glycerophosphate bond and remove the base group of phospholipids, respectively. While a major role of phospholipase function is membrane homeostasis, additional functions comprise nutrient digestion and generation of signaling molecules. Some phospholipases are toxins or components of venoms. Bacterial phospholipases have been shown to be involved in pathogenesis by promoting hemolysis, cytolysis, and tissue destruction, as well as interfering with host signal transduction [78].

As indicated in Table 8, the largest and best-characterized group of phospholipases in *C. albicans* is the five-member phospholipase B gene family. A related gene family is present in *S. cerevisiae,* albeit with three members, again reflecting the general increase in gene numbers for enzymes involved in lipid metabolism in *C. albicans*. All PLB proteins harbor NH_2_-terminal signal peptides for secretion; Plb3p, Plb4p, and Plb5p additionally contain hydrophobic COOH termini with putative GPI anchor attachment sites for localization to the plasma membrane or further processing for tethering to the cell wall [79,80]. To date, *PLB1* and *PLB2* are the best-characterized members of the gene family [81–84]. Inactivation of *PLB1* [82,83] and *PLB5* (S. Theiss, G. Ishdorj, M. Kretschman, C. Y. Lan, T. Nichterlein, et al., unpublished data) reduced virulence in animal models.

**Table 8:**
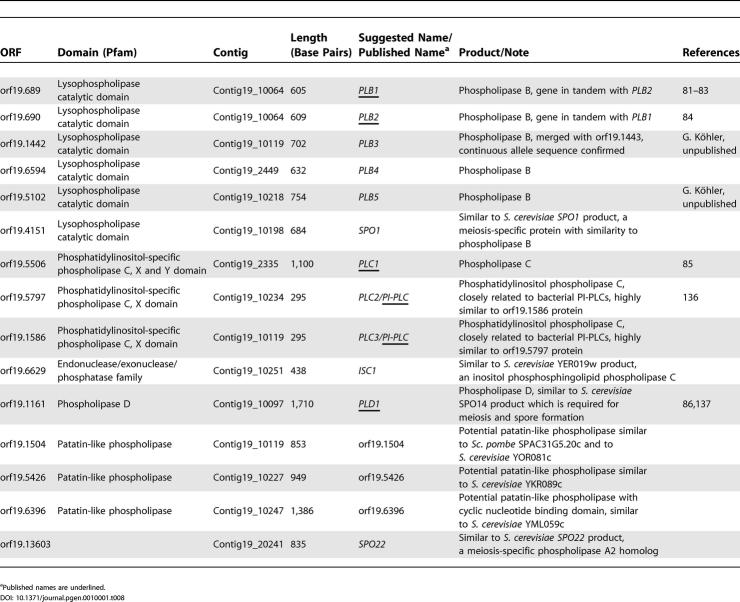
Phospholipases in *C. albicans*

^a^Published names are underlined.

Putative PLC and PLD phosphodiesterases are also represented in the *C. albicans* genome. Orf19.6629 is a likely homolog to *S. cerevisiae* *ScISC1,* which encodes a PLC with neutral sphingomyelinase activity. Besides the recently published *PLC1* gene [85], two almost identical genes encode phosphatidylinositol phospholipase C proteins (PI-PLC). The latter lack homologs in *S. cerevisiae,* but are similar to bacterial PI-PLCs. *PLD1* was shown to be involved in the morphological transition from yeast to hyphae and required for full virulence in animal models [86]. Interestingly, the *PLD1* gene product and another phospholipase-like protein (encoded by orf19.4151) show significant sequence similarity to *S. cerevisiae* proteins that are involved in meiosis and sporulation (ScSpo1p, ScSpo14p, and ScSpo22p). As already shown for *PLD1,* the *ScSPO1* homolog, the functional roles of these proteins are likely to differ from their counterparts in *S. cerevisiae* since *C. albicans* has not been shown to undergo meiosis [10].

Another intriguing group of phospholipase genes in *C. albicans* are patatin-like phospholipases encoded by orf19.1504, orf19.5426, and orf19.6396. These proteins might account for phospholipase A activities in *C. albicans* that could be involved in intracellular storage or mobilization of lipids.

#### Sphingolipid metabolism.

*C. albicans* also displays differences from *S. cerevisiae* with respect to sphingolipid metabolism. Pathways leading to and from fungal-type sphingomyelins have been studied extensively in *S. cerevisiae,* where by-products mediate many important structural and signaling functions that affect cell proliferation, the definition of cell membrane domains and polarity, apoptosis, and stress responses [87,88]. Many of the associated enzymes are essential and are targets of fungal toxins, and thus are candidates for anti-fungal drug development [88]. *C. albicans* shares the same fundamental pathways in sphingolipid biosynthesis/degradation plus four additional enzymes. Two of these, a glucosyl transferase (CGT1) and a delta-4 sphingolipid desaturase (DES1), have been previously studied. The presence of glycosyl ceramides in *C. albicans* has been known for some time [89,90], and the gene responsible for their synthesis has been cloned and expressed in *Pichia* [91]. The molecules play a common role in differentiation in dimorphic fungi [92]. Homologs of the delta-4 sphingolipid desaturase enzyme include the mouse, human, and *Drosophila* degenerative spermatocyte proteins, which play a role in meiosis [93]; its function in *C. albicans* may relate to membrane structure, or the production of signaling molecules, as is the case in plants. An interesting component of sphingolipid metabolism in *C. albicans* is a sphingomyelin transfer protein (Het1p) similar to the *Podospora anserina* HET-C2 protein. The *P. anserina* protein is involved in self/non-self discrimination, a fungal version of the vertebrate major histocompatibility locus [94,95]. It is possible that the protein is involved in regulating the sphingomyelin composition of *C. albicans* membranes, a factor that may relate to acquisition of resistance to amphotericin B and azoles [96]. Finally, *C. albicans* encodes four acid sphingomyelinases, two of which may be secreted, that have not been studied in fungi. Based on the actions of metazoan secreted acid sphingomyelinases, these enzymes may be involved in regulation of membrane raft formation and generation of ceramide, a second messenger that is known to regulate apoptosis in higher eukaryotes. Secreted sphingomyelinases of pathogenic bacteria, which are enzymatically similar but structurally unrelated to those of *C. albicans,* have been shown to lyse phagosomal membranes [97], facilitate entry into both phagocytic and nonphagocytic cells [98,99], act as hemolysins that abet piracy of iron from the host [100,101], and induce host cell apoptosis [102,103].

### Signal Transduction

Differences in signal transduction and regulatory pathways between *C. albicans* and *S. cerevisiae* are numerous. Many of these *C. albicans*–specific genes encode proteins that are responsive to changes in the environment. They may thus be responsive to colonization of a new anatomical site (e.g., passage through the stomach), fluctuations in the availability of nutrients, or the appearance of host inflammatory reactions. Gene products falling into this category include (1) a homolog (TIP120) of a TBP-interacting protein in humans and rats, which acts as global regulator of class I, II, and III genes in response to abrupt changes in ambient conditions [104], (2) a relative (orf 19.1798) of tuberin, a negative regulator of cell growth in response to low cellular energy levels in mammals [105], (3) a conserved group of stomatin-like proteins (orf 19.7296 and SLP2) that may play a role in mechanoreception, (4) a family of pirin homologs that obviously arose from a recent duplication event *(PRN1, PRN2, PRN3,* and *PRN4)—*these are nuclear factors whose homologs interact with the human oncogene Bcl-3 product and with an *A. thaliana* G protein alpha-subunit involved in regulating seed germination and early seedling development [106])—and (5) a rhomboid protein (orf 19.5234), probably located on the plasma membrane, whose homologs in eukaryotes and bacteria mediate the proteolytic release of signaling peptides from a larger precursor [107]. In addition to differences traceable to novel genes, other pathways that share components have doubtless been altered in their role and regulation, such as the mating pathway [108].

Two of the most important enzyme families that are involved in signal transduction pathways are the kinases and small GTPases. The *C. albicans* annotation identifies 96 protein kinases, most of which have strong orthologs in *S. cerevisiae*. The *C. albicans* genome contains two genes encoding GTPases of the heterotrimeric G protein alpha-subunit family—*GPA1* and *GPA2*. In addition, it contains 29 small GTPases of the p21 superfamily. These include a single Ras protein (Ras1p), various members of the Rho and Rab families, the Ran1 homolog Gsp1p, and several members of the ADP ribosylation subfamily. Most of these proteins have clear *S. cerevisiae* orthologs. However, *S. cerevisiae* does not have a Rac homolog, while orf19.6237 appears to encode a *C. albicans* Rac protein and has thus been named *RAC1*. As well, orf19.5902 appears to be distantly related to Ras but lacks any strong equivalent in any organism, and has been designated Rlp1p, for Ras-like protein, while orf19.2975 is a YPT/RAB family member that has been named *RAB7* because it has no clear *S. cerevisiae* YPT ortholog.

### Conclusions

We have coordinated a community-wide effort to manually confirm, edit, and annotate 6,354 genes from assembly 19 of the *C. albicans* genome. This annotation includes 214 intron-containing genes, 246 genes with either missense mutations or sequencing errors, and 190 truncated genes that terminate at the ends of the sequence contigs. *C. albicans* genes were found to be exceptionally rich in short sequence repeats, especially compared to the genomes of *S. pombe* and *S. cerevisiae*. Correlation with transcriptional profiling data was used to identify potentially spurious genes. This improved dataset allowed the identification fungal-specific genes and permitted a detailed analysis of several large multigene families. Comparative genomic studies indicate that *C. albicans* is much more versatile in its production of secreted lipid- and amino-acid-degrading enzymes and in its ability to import the resulting nutrients.

## Materials and Methods

### 

#### Identification of *C. albicans* ORFs and merging of preliminary annotations.

Nucleotide sequence data for assembly 19 were retrieved from the SGTC Web site (http://www-sequence.stanford.edu//group/candida/). Assembly 19 is composed of a haploid supercontig set (contigs 19–831 to 19–10262), here referred to as the haploid set, and a allelic supercontig set (contigs 19–20001 to 19–20161), here referred to as the allelic set [6].

The CAAT-Box software package [109] was used to identify annotation-relevant ORFs in assembly orf19. A set including ORFs longer than 300 codons and a set with all intergenic regions obtained after subtraction of ORFs larger than 80 codons were created. These sets were used to build a GeneMark matrix [11] that was subsequently used to evaluate the coding probability of all ORFs in assembly 19. ORFs longer than 150 codons, and ORFs longer than 40 codons and with a GeneMark coding function greater than 0.5 [11] over their whole length, were selected and assigned a reference number of the format IPF*n.i* where IPF stands for individual protein file, *n* is an integer specific to the IPF, and *i* corresponds to the number of times the IPF has been modified between assembly 5, 6, and 19 of the *C. albicans* genome sequence. In total, 11,025 and 9,089 IPFs were selected in the haploid and allelic sets, respectively. IPFs shorter than 150 codons in the haploid set were further inspected for (1) overlaps with larger IPFs on a different frame and (2) homology to proteins in the NR database of non-redundant proteins from GenBank. IPFs that overlapped with a larger IPF or did not show a significant homolog (BLASTP *e*-value < 1e^−3^) [110] were designated FALSORF. Of the 11,025 IPFs identified in the haploid set, 3,505 were FALSORFs.

All IPFs identified in the haploid set were compared through reciprocal BLASTP to the set of 7,680 *C. albicans* ORFs defined at the SGTC that uses the systematic designation orf19.*n.* BLASTP results were parsed using Readblast [111]. IPFs without an orf19.*n* counterpart were assigned a new reference number of the format orf19.*n.i,* where orf19.*n* is the closest upstream (using SGTC contig coordinates) ORF defined by the SGTC and *i* is an integer that varies between 1 and the number of IPFs located between orf19.*n* and orf19.(*n* + 1). For instance, if three ORFs were found between orf19.1234 and orf19.1235, these would be referred to as orf19.1234.1, orf19.1234.2, and orf19.1234.3. Taken together, 11,616 orf19 ORFs were identified in the haploid set, of which 3,936 were not present in the SGTC orf19 set.

A similar procedure was applied to the allelic set of sequences, and a total of 9,552 orf19 ORFs were identified, of which 3,012 were not present in the SGTC orf19 set. The haploid and allelic sets of orf19 ORFs were compared by reciprocal BLASTP in order to define allelic and unique sequences in the allelic set (see below).

The 11,615 orf19 ORFs identified in the haploid set were compared by reciprocal BLASTP to the 9,168 ORFs identified by the SGTC using assembly 6 of the *C. albicans* genome sequence (designated orf6.*n*). A similar reciprocal comparison was run using the set of 6,165 *C. albicans* proteins available in the CandidaDB database that have been defined by applying a procedure similar to that outlined above on assembly 6 and through a manual curation aiming to reach a non-redundant protein set (http://genolist.pasteur.fr/CandidaDB; [12]). Furthermore, orf19 ORFs were reciprocally compared to the *S. cerevisiae* proteome using data available at the SGD [112]. All data were parsed using Readblast [111], and a matrix was generated that correlated ORFs from each dataset.

We used the genome annotation tool Artemis [14], which provides very detailed annotation capability, visually mapping desired features onto the target sequence. In preparation, we loaded our heterogeneous data into the required EMBL-style files: (1) the orf19 reference (field: Assembly_ID); (2) the orf6 reference (field: old_Assembly_ID); (3) the CandidaDB entry number (field: db_xref); (4) the entry number for the Comprehensive Yeast Genome Database, which provides a detailed analysis of protein features of all entries available in CandidaDB [113] (field: db_xref); (5) the GenBank entry number for *C. albicans* proteins previously characterized and annotated (field: db_xref); (6) the IPF reference (field: db_xref); (7) annotation data available from CandidaDB (proposed gene name and proposed function; field: Annotator); (8) annotation data of the Agabian's laboratory based on the orf6 protein set (http://agabian.ucsf.edu/canoDB/anno.php) (field: Annotator); (9) annotation data of the Fink's and Johnson's laboratories based on the orf6 protein set (unpublished) (field: note_AJ); (10) annotation comments available from CandidaDB (field: note_GF); (11) Pfam matches [114] obtained using the orf19 protein set (field: pfam_match); (12) Clusters of Orthologous Groups matches [115] obtained from the Comprehensive Yeast Genome Database using the CandidaDB protein set (field: COGs_MIPS); (13) EC number matches obtained from the Comprehensive Yeast Genome Database using the CandidaDB protein set (field: EC_number_MIPS); (14) *S. cerevisiae* closest protein including *e*-value, putative orthology, protein function, gene name, and alternate gene names, genome reference number obtained from SGD (field: Note); (15) GO annotation of the *S. cerevisiae* protein obtained from SGD (field: GO); (16) the reference of the allelic orf19 in the allelic set of sequences including supercontig number and location (field: Allele); and (17) chromosome assignment data available from the Magee's and Whiteway's laboratories (http://206.167.190.233/candida/index.cfm?page=CaChrom) (field: Chromosome).

Furthermore all ORFs were assigned a color code in order to facilitate annotation using the annotation tool Artemis [14]. All orf19 ORFs corresponding to an IPF classified as FALSORF and orf19 ORFs identified at the SGTC and not found among IPFs were color-coded in grey. orf19 ORFs with an unambiguous allele (90% identical amino acids over the whole length of the longest ORF) were color-coded in red (>150 codons) or pink (<150 codons). orf19 ORFs with a questionable allele (90% identical amino acids over the whole length of the shortest ORF) were color-coded in green (>150 codons) or pale green (<150 codons). orf19 ORFs without a clear allele (less than 90% identical amino acids over the whole length of the shortest ORF or no reciprocal match) were color-coded in blue (>150 codons) or light blue (<150 codons).

From this “master” file of sequences and their corresponding preliminary annotation, groups of contigs were selected and saved as partially annotated subsequences that were reserved and retrieved by members of the annotation consortium, and once fully annotated, were returned to a central Web site. A version of Artemis was distributed to the consortium that included a modified “options” file [116] allowing project-specific qualifiers to be used, and also featuring the *C. albicans*–specific translation Table 12 [117].

#### Whole genome BLAST searches and visualization of sequence homologies.

ORF sequences were translated to proteins using the translation table for *C. albicans* [117], and compared using the BLASTP algorithm [118] with the NR database, the *C. albicans* proteome itself, the putative proteomes of five fungi *(S. cerevisiae, S. pombe, N. crassa,* *A. nidulans,* and *M. grisea),* and the proteomes of five other eukaryotes *(A. thaliana, D. melanogaster, C. elegans, M. musculus,* and *H. sapiens)*. Sequence data were obtained from the EMBL-EBI Integr8 Browser (http://www.ebi.ac.uk/integr8/EBI-Integr8-HomePage. do) and the Broad Institute for Genome Research (http://www.broad. mit.edu/annotation/). For the most similar proteins by BLAST, negative exponents of the *e-*values were parsed from the output files, collected in a relational database, and visualized as a color range associated with each reference protein (see Figure 1). For this purpose, a Web-accessible visualization tool was constructed using Macromedia Cold Fusion and an Apache2 Web server. These results can be consulted at For this purpose, a Web-accessible visualization tool was constructed using Maromedia Cold Fusion and an Apache2 Web server. These results can be consulted at http://candida.bri.nrc.ca/candida/index.cfm?page =blast.

#### Bioinformatic identification of putative introns.

Intron locations were predicted by constructing regular expressions based on consensus data drawn from several known introns in *C. albicans* (SOD1, EFB1, CMD1, CSK1, and others) as well as the extensive knowledge of the splice site consensus in *S. cerevisiae* [119] and matching of those expressions to the genomic DNA. Two regular expressions were used: /(.{15,}?TG[AT]A[CT]G)/ and /(.{700}) ([ATC]TACTAAC.{4,24}?[ATC][CTA]AG)(.{90})/. These incorporate several conserved aspects of mRNA splice sites including (1) the splice donor site of G(T/C)A(T/A)GT, where the initial guanine is the first residue within the intron, (2) a gap of between 15 and 700 nucleotides between the donor site and the branch point, (3) the branch point consensus sequence (A/T/C)TACTAAC, (4) a gap of between four and 24 nucleotides between the branch point and the splice acceptor site, (5) the splice acceptor site (T/A/C)(T/A/C)AG, where the final guanine is the final residue within the intron. This procedure provided landmarks that annotators could use to decide whether a gene contained introns, a decision also based on the position of nearby ORFs, on the ability of the intron/exon assembly to extend the size of the reading frame, and, most important, on the ability of the putative intron/exon assembly to improve similarity to sequences in other species. It predicted 1,297 introns in the *C. albicans* genome, many of which were not near ORFs or were otherwise incorrect. Nevertheless, this procedure was useful in providing markers for confirmation by the annotators.

#### Identification and classification of STRs.

A STR was defined as a short nucleotide element (1–20 nt) repeated a number of times with a periodicity *P,* a length *L,* and a tolerated mutation window *W*. Since allelic indels are rare in *C. albicans,* we considered only mutations in STR sequences and not insertion or deletion from the consensus periodic pattern. *W* indicates the minimun distance between two mutations within a STR. For example, the STR “CTACAACAACAGCAAC” has *P* = 3, *L* = 16, and *W* ≤ 10. When two STRs overlap they are merged together defining a single STR domain. Since short STRs can randomly occur, depending on the G/C content of the genome, we established a significant threshold length *L*_MIN_ for every periodicity *P* and mutation window *W* to represent the STR length for which the number of STR domains found in a randomized genome is less than 5% of the number found in the real genome. We call STR95 the set of all STR domains that are longer than the threshold value. In the STR95 set, every STR domain has a less than 5% chance of being a random event. With this method of setting the minimal STR length, no significant STR can be found in any randomized genome or in genomes that do not contain STRs in sufficient number to beat the odds 20 to one. More information and data can be found in Dataset S6.

#### Identification of spurious genes.

For the calculation of gene expression correlation, we used a set of approximately 1,000 *S. cerevisiae* microarray experiments [127], and 216 genome-wide *C. albicans* expression profiles [7,13,108,120–126]. The iterative signature algorithm [35,127] was applied to the *C. albicans* expression data as described. We analyzed ORFs present on the arrays for which an orf19 number could be determined, including some that were subsequently removed from the final set of annotated genes. Pairwise Pearson correlation coefficients were calculated for each ORF with respect to all other ORFs across all of the experiments contained in the dataset. Random subsets of the *S. cerevisiae* data were generated by randomly selecting 200 experiments from the complete set of approximately 1,000 profiles. ORFs whose correlation coefficient exceeded the threshold value of 3σ with at least one other ORF in the dataset were recorded and excluded from the list of spurious gene candidates. The standard deviation σ of the background correlation of random gene pairs was measured to be σ = 0.16 for *S. cerevisiae* and σ = 0.21 for *C. albicans*. Similarly, genes possessing an ortholog in *S. cerevisiae* were excluded from the list of *C. albicans* candidates, and vice versa. All remaining ORFs were subsequently ordered by their length, and the 50 shortest ORFs were excluded (many of the shortest 50 genes correspond to real genes in *S. cerevisiae*).

## Supporting Information

Dataset S1 Coordinates and All of the Annotation Fields for the 6,354 Confirmed *C. albicans* Genes, Based on the Version 19 Genome Assembly Please note that Microsoft Excel may convert some of the gene names to dates and fail to import some of the largest fields.
(2 MB TXT)

Dataset S2 Sequence and Position of All Statistically Significant STRs in *C. albicans* Coding Sequences (291 KB TXT)

Dataset S3 Sequence and Position of All Statistically Significant STRs in *S. pombe* Coding Sequences (11 KB TXT)

Dataset S4 Sequence and Position of All Statistically Significant STRs in *S. cerevisiae* Coding Sequences (66 KB TXT)

Dataset S5 Sequence and Position of All Statistically Significant STRs in *N. crassa* Coding Sequences (488 KB TXT)

Dataset S6 Detailed Description of Our STR Identification Algorithm (4 KB TXT)

Table S1 List of Potentially Spurious Genes (34 KB XLS)

### Accession Numbers

The GenBank (http://www.ncbi.nlm.nih.gov/Genbank/) accession numbers for ORFs discussed in this paper are *ALS3–1* (AY223552), *ALS3–2* (AY223551), *ALS4–2* (AF272027), *ALS5–1* (AY227440), *ALS5–2* (AY227439), strain SC5314 sequence corresponding to *ALS6* (AY225310), strain SC5314 *ALS9–1* (AY269423), and strain SC5314 *ALS9–2* (AY269422).

## Abbreviations

ABC: ATP-binding cassette
CGD: *Candida* Genome Database
*e*-value: expect value
EC: Enzyme Commission
GO: Gene Ontology
IPF: individual protein file
NR: non-redundant
ORF: open reading frame
SGD: *Saccharomyces* Genome Database
SGTC: Stanford Genome Technology Center
STR: short tandem repeat
TMS: transmembrane segment
